# Interventional radiology and artificial intelligence in radiology: Is it time to enhance the vision of our medical students?

**DOI:** 10.1186/s13244-020-00942-y

**Published:** 2020-11-30

**Authors:** Pierre Auloge, Julien Garnon, Joey Marie Robinson, Sarah Dbouk, Jean Sibilia, Marc Braun, Dominique Vanpee, Guillaume Koch, Roberto Luigi Cazzato, Afshin Gangi

**Affiliations:** 1 Interventional Radiology, CHRU Strasbourg, 1, Place de l’Hôpital, B.P. 426, 67091 Strasbourg Cedex, France; 2 Rheumatology Department, CHRU Strasbourg, 1 Avenue Molière, 67200 Strasbourg, France; 3 Diagnostic and Interventional Neuroradiology, CHRU Nancy, 29 Avenue du Maréchal de Lattre de Tassigny, 54035 Nancy, France; 4 IADI - Inserm 1254, Université de Lorraine, Nancy, France; 5 Institut de Recherche en Santé Et Société, Faculté de Médecine, Université Catholique de Louvain, Clos Chapelle-aux-champs 30/B1.30.16, 1200 Woluwe-Saint-Lambert, Belgium

**Keywords:** Radiology, Interventional radiology, Artificial intelligence, Education, Female

## Abstract

**Objectives:**

To assess awareness and knowledge of Interventional Radiology (IR) in a large population of medical students in 2019.

**Methods:**

An anonymous survey was distributed electronically to 9546 medical students from first to sixth year at three European medical schools. The survey contained 14 questions, including two general questions on diagnostic radiology (DR) and artificial intelligence (AI), and 11 on IR. Responses were analyzed for all students and compared between preclinical (PCs) (first to third year) and clinical phase (Cs) (fourth to sixth year) of medical school. Of 9546 students, 1459 students (15.3%) answered the survey.

**Results:**

On DR questions, 34.8% answered that AI is a threat for radiologists (PCs: 246/725 (33.9%); Cs: 248/734 (36%)) and 91.1% thought that radiology has a future (PCs: 668/725 (92.1%); Cs: 657/734 (89.5%)). On IR questions, 80.8% (1179/1459) students had already heard of IR; 75.7% (1104/1459) stated that their knowledge of IR wasn’t as good as the other specialties and 80% would like more lectures on IR. Finally, 24.2% (353/1459) indicated an interest in a career in IR with a majority of women in preclinical phase, but this trend reverses in clinical phase.

**Conclusions:**

Development of new technology supporting advances in artificial intelligence will likely continue to change the landscape of radiology; however, medical students remain confident in the need for specialty-trained human physicians in the future of radiology as a clinical practice. A large majority of medical students would like more information about IR in their medical curriculum; almost a quarter of students would be interested in a career in IR.

## Key points

- New technologies and other advances, including artificial intelligence, will probably change the landscape of Radiology; however, medical students remain confident in the future of Radiology.
- A large majority of medical students would like more information/lectures about Interventional radiology in their medical curriculum; almost a quarter of students would be interested in a career in IR, which is very promising for the future of this specialty.
- The organization of the specialty and access to academic positions must evolve to be more attractive to women, who remain under-represented in interventional radiology.

## Introduction

Radiology has long been a very attractive specialty, widely chosen by medical students in the first preference specialties for residency. It serves an essential aspect of patient care, enabling more accurate diagnoses more quickly, and assessment of the effects of treatments, to support effective treatment management. The popularity of diagnostic radiology was sustained even as the interventional branch of radiology began to develop decades ago. Interventional radiology (IR) has undergone major growth in the last two decades, thanks in large part to the substantive advances in its core technologies. Interventional radiologists utilize image guidance to navigate minimally invasive routes to treat a wide variety of pathologies (oncologic, pain, fracture, hemorrhagia, ischemia, and numerous other neurovascular and endovascular disorders), often resulting in a faster recovery than open surgery [1, 2] and enabling treatment for patients with important comorbidities who might not be candidates for surgery or other treatment modalities.

However, in recent years, technological innovations supporting the development of artificial intelligence and teleradiology have generated speculation about the future of radiology, both diagnostic and interventional, and raised doubts about the longer-term viability as a clinical practice. As a large teaching institution, we wanted to assess students' thoughts about the future of radiology and how they perceive artificial intelligence and its role vis-à-vis radiology. Moreover, we wanted to determine the awareness and knowledge of IR among medical students at different phases of their study.

One element of our interest in this study is the shift in categorization of interventional radiology as a clinical practice. As it developed out of the well-established field of radiology, IR evolved as a subspecialty of radiology: medical students still choose radiology for their residency and engage in further years of specialty interventional training if they choose an IR path for “added qualifications.” In some countries, such as France, an option during the radiology internship now allows a path of special training in IR during their last two years of internship. However, 2020 is a year of educational shift in the USA, as the medical school and training definition of IR in the USA is evolving to reflect the advancing role of IR across clinical arenas. As of June 2020, US medical students may choose IR directly, as a specialty for their residency and intended future practice [1]. The implementation of this represents a shift not only for those students making such a decision, but in medical school operational planning. So in that regard, we find further value in quantifying the thoughts and attitudes of medical students toward the future of IR and how they perceive the evolution of radiology with the developments of artificial intelligence.

## Materials and methods

### Study design

The French data protection authority confirmed permission to send the survey to European students and collect anonymous data. Ten medical schools in eight European countries and one medical school in the USA were invited to participate to the survey. Three EU medical schools accepted and sent the survey to students (medical school of Strasbourg, medical school of Nancy, and Catholic University of Louvain). Four medical schools did not respond, two declined to send the survey, and two accepted but did not send the survey to students. The two medical schools that declined to participate in the survey cited data protection laws, despite the anonymous basis of the survey. Among the three medical schools which accepted and sent the survey, medical students had between 2 and 4 h per year of dedicated IR lectures during the clinical phase (4th to 6th years) and no dedicated IR lectures during preclinical phase.

Between January and June 2019, deans of medical schools were contacted and the electronic survey was sent by email to the Dean’s secretary who then forward to all medical students from first to sixth year of the three medical schools that agreed to participate. This survey was sent to 9546 medical students and contained two category questions and 14 content questions: two related to Radiology in general and 12 related to IR (Appendix). Responses were collected online in a secure, dedicated platform (Google forms). Participation was voluntary and anonymous.

### Electronic survey

Question 1 was the year of medical school of the student, which allowed grouping the students in two categories: preclinical (from first to third years) and clinical (from fourth to sixth years). Question 2 was gender (male or female) to assess any difference between genders. Questions 3 and 4 deal with artificial intelligence and its role in the future of radiology. The remaining questions were specific to IR. Questions 5 to 11 assess students' knowledge about interventional radiology. Then the two last questions asked the students if he would be interested in a career in IR and the reasons if doesn’t want to.

### Data collection and analysis

All data were collected anonymously on an Excel files (Microsoft). These data were analyzed for all students of the three medical schools, and a subgroup analysis was performed between preclinical and clinical groups.

### Statistical analysis

Categorical variables are provided as absolute number and percentage. Categorical data were tested with Chi square or Fischer exact test. *p* < 0.05 was considered statistically significant. Statistical analysis was performed using SAS version 9.4 (SAS, Cary, NC).

## Results

### Questions 1 and 2: Study year and gender:

**Fig. 1:**
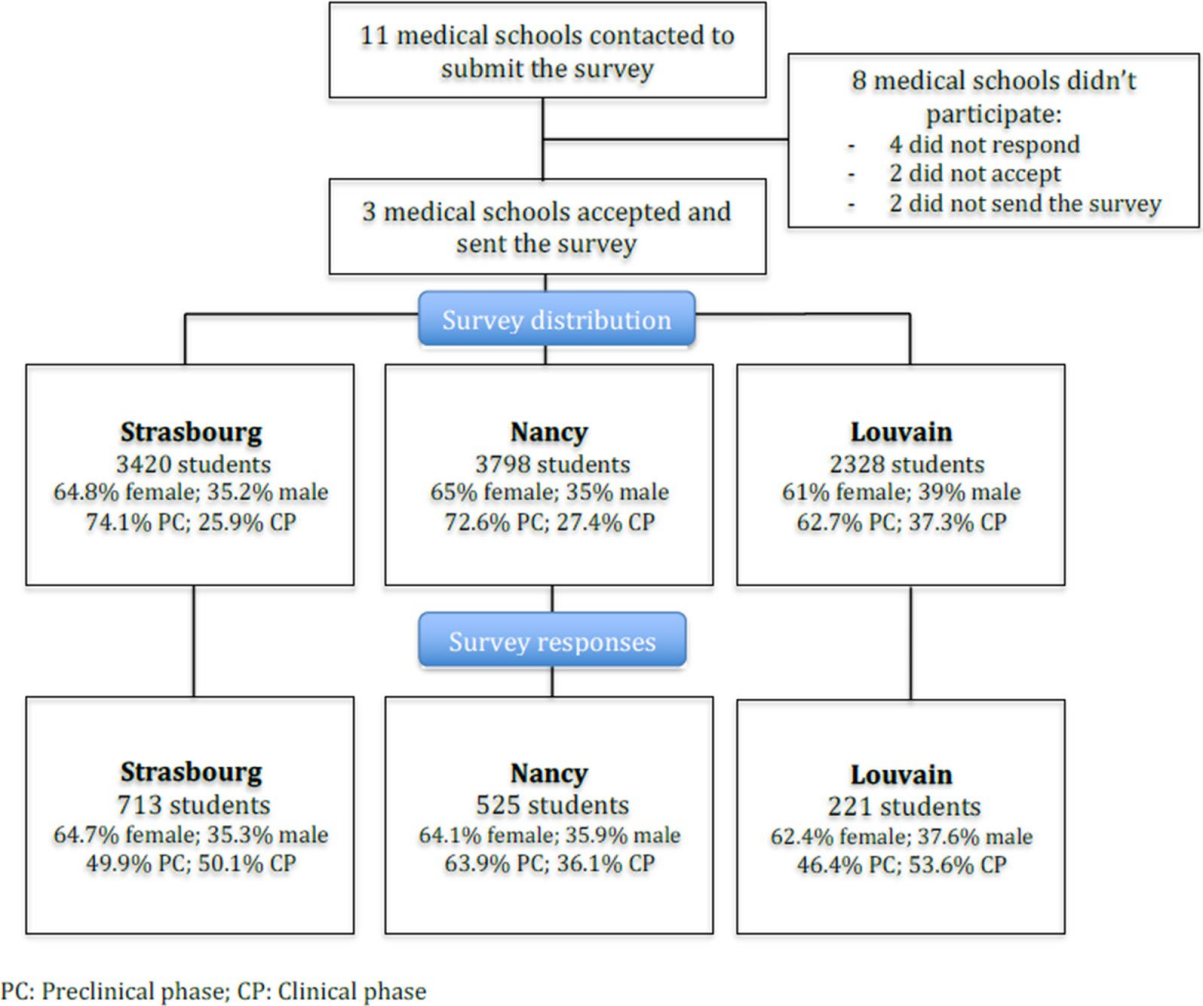
Flowcharts illustrating the distribution of students by medical school. PC: preclinical phase; CP: clinical phase

Of the 9,546 students who received the survey (17.4%, CI 95%: 20.2–24.4), 1459 students responded (15.3%): 713 from Strasbourg (64.7% female, 35.3% male), 525 from Nancy (65% female, 35% male), and 221 from Louvain (62.4% female, 37.6% male) (Fig. 1).

The distribution of the students shows that 26% were in the first year, 11% in the second year, 13% in the third year, 14% in the fourth year, 16% in the fifth year and 20% in the sixth year (preclinical phase: 50.3% and clinical phase: 49.7%), with a gender ratio M/F of 2/3: 37.4% were male and 63.6% were female (preclinical phase: male: 36%, female: 34%; clinical phase: male: 38%, female:62%; *p* = 0.23).

### Questions 3–4: Radiologists and radiology

Thirty five percent (CI95%: 32.5–37.4) of all students think that artificial intelligence is a threat to radiologists (preclinical phase: 33.9%; clinical phase: 34%; *p* = 0.44).

Ninety one percent (CI 95%: 89.5–92.4) of all students think that radiology has a future (preclinical phase: 92%; clinical phase: 89%; *p* = 0.11).

### Questions 5–14: Interventional radiology—knowledge and interest

Eighty-one percent (CI 95%: 80–83%) of all students have ever heard of interventional radiology (preclinical phase: 64%, clinical phase: 96%; *p* = 0.003^–46^). Major part of the students have heard about interventional radiology: 47.4% (CI 95%: 44.5–50.2) during a lecture; 30.7% (CI 95%: 28.1–33.3) from general reading, 12.1% (CI 95%: 10.2–14) from family, general reading, or patients and 9.8% (CI 95%: 8.1–11.5) from clinical attachment.

Twenty-eight percent (CI 95%: 25.7–30.3) of all respondents have ever had a lecture on interventional radiology (preclinical phase: 22%; clinical phase: 32%, *p* = 0.0008).

Eighty percent (CI 95%: 77.9–82) of all students would like more formal lecture and/or information about interventional radiology (preclinical: 83.5%; clinical: 75.9%, *p* = 0.001).

The self-evaluation of their knowledge in interventional radiology compared to the other specialties shows that 0.3% consider their knowledge as excellent, 2.6% as good, 20.6% as adequate, 56.4% as poor and 20.1% as no knowledge (preclinical phase: Excellent: 0.2%, Good: 1.8%, Adequate: 20.6%, Poor: 44.5%, No Knowledge: 32.9%; clinical phase: Excellent: 0.1%, Good: 3.5%, Adequate: 24.7%, Poor: 65.4%, No Knowledge: 6.3%).

Eighty eight percent (CI 95% = 86.3–89.7) of students do not know how to become an interventional radiologist (preclinical phase: 95.2%; clinical phase = 80.7%, *p* = 1.6 E−14).

Fifty-one percent (CI 95%: 48.4–53.6) think that interventional radiology is surgery guided by imaging like CT, MRI and Ultrasound, 30% were not sure and 19% think that IR is not surgery guided by imaging modalities (preclinical phase: Yes: 53%, Not sure: 38%, No: 9%; clinical phase: Yes: 49%, Not sure: 23%, No: 28%; *p* = 5.68E−19).

**Fig. 2:**
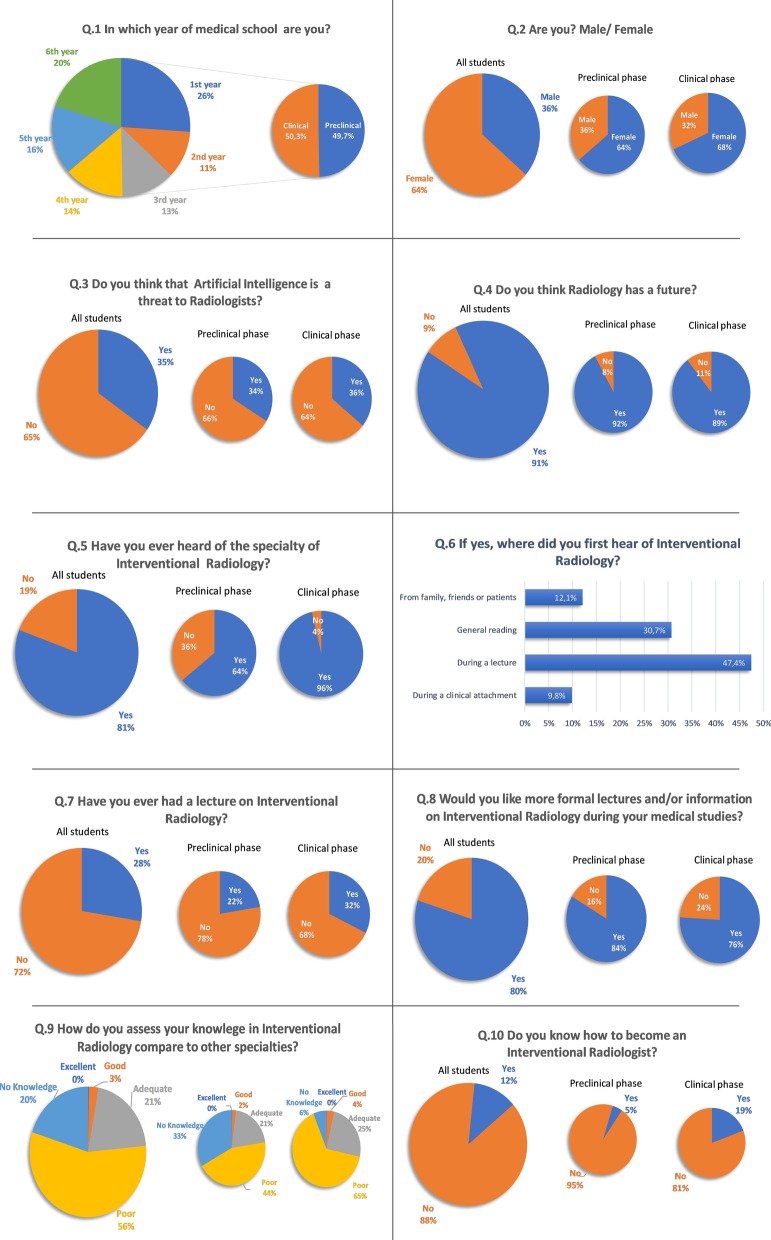

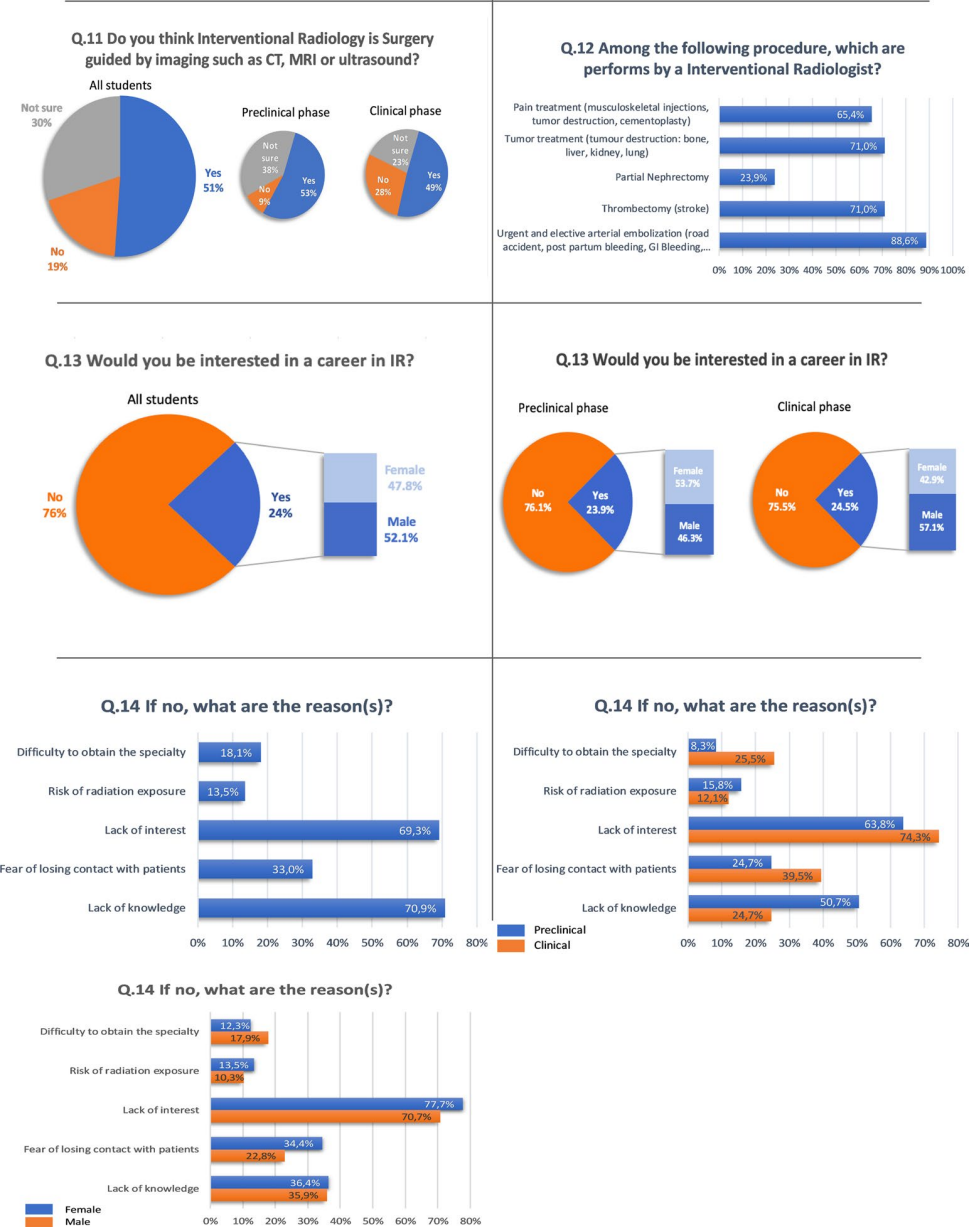
Survey results

65.4% of students think that pain treatment is in the field of IR, 71% think that tumor treatment is performed by interventional radiologists, 23.9% think that partial nephrectomy is practice by interventional radiologist, 71% think that thrombectomy is performed by interventional radiologist and 88.6% think that urgent and elective arterial embolization were done by interventional radiologist (Fig. 2).

Twenty-four percent of all students would be interested in a career in interventional radiology, 47.8% of which are female and 52.1% male (preclinical phase: 23.9% of which 53.7% female and 46.3% male; clinical phase: 24.5% of which 42.9% female and 57.1% male; *p* = 0.79).

Among students who are not interested in a career in IR, 70.1% selected a lack of knowledge, 69.3% selected lack of interest, 33% selected the fear of losing contact with patients, 18.1% selected the difficulty in obtaining the specialty and/or 13.5% selected the risk of radiation exposure.

## Discussion

Recent technological advances, including applications of artificial intelligence, have fueled speculation about the future of diagnostic radiology. In 2016, an oncologist predicted that “machine learning will shift much of the work of radiologists and pathologists” [3]. The founder of Google Brain Deep learning, Andrew Ng, said in the Economist that radiologists would be replaced by AI sooner than their executive assistants [4]. Their enthusiastic statements gave medical students food for thought about their future specialty. Despite the subsequent revision of the experts' thinking [5–7], the impact has endured, causing mistrust among medical students of the changes that will result from these new technologies. Since the promotion of AI in radiology, the student ranking choosing radiology as a specialty in France has declined. Thus, radiology, which was the most attractive specialty when residents made their choice of specialty in France in 2012, was only in 7th position in 2017 when comparing attractiveness indices [8, 9]. Indeed, our study shows that more than a third of the students think that AI is a threat to radiologists, with no significant shift in opinion between the students’ preclinical and clinical phases of study. It shows that the information given to medical students regarding artificial intelligence and its possibilities is not keeping pace with technological advances. However, AI specialists and radiologists work, and will continue to work, together to develop software to advance the profession and optimize outcomes. AI is evidently a game-changer for the future of radiology; recent publications have touted the superior accuracy of AI over human diagnostic specialists in detection of certain cancers [10, 11].

More than 90% of students in our study expressed a belief that radiology has a future. These results confirmed the previous results reported by Pinto dos Santos et al., wherein 83% of medical students disagreed with statements that human radiologists would be replaced by AI [12]. Indeed, as Prof. Langlotz explains in a recent publication, AI will help radiologists and complement their skills [13]. Thus, the purpose of AI is not to replace the radiologist; it is a tool to augment capabilities and improve outcomes across multiple specialties. And probably, as Blum et al. said, the radiologist's main enemy is not the AI but the radiologist himself, and he needs to focus more on the patient and invest in examining and understanding clinics in order to value his work [14]. As a clinical practice, interventional radiology is a marriage of diagnosis and treatment. In addition, for therapeutic interventions, patient follow-up is carried out by the IR who treats the patient and who therefore understands the pathology presented by the patient. Interventional radiology has experienced significant growth in recent years; our study shows widespread awareness of the specialty among medical students (81%). This result is in line with the previous study of De Gregorio et al. which reported 72.8% awareness by medical students [15]. A large proportion of students have heard about IR in a lecture but more than two-thirds have never had a lecture *dedicated to IR.* This means that students hear about interventional radiology during courses done by the other specialties. The study highlights the lack of courses on IR in the medical curriculum at any phase of medical school: 73% of sixth year students in our study had not had a course on IR. This is troubling on multiple levels students cannot know they have an interest in pursuing a specialty they are unaware of; without awareness of IR and its place in patient care, residents and early MDs in general practice or other specialties cannot provide the full array of options to their patients who may benefit from an IR therapy. Eighty percent of the students in our study would like more courses on IR, which is close to the results of de Gregorio's study which reported that more than 99% of medical students wanted more information on IR [15]. Therefore, IRs need to be more present in their universities to share their knowledge of IR, and its place in medical care, with medical students. There are already lectures on diagnostic radiology in medical schools; lectures specific to interventional radiology should be an integrated part of the curriculum. Indeed, the self-assessment by medical students of their knowledge of IR compared to other specialties is worrisome. These same results were also reported in previous studies in Europe and Canada with a clear majority of students (between 55.9% and 66%) rating their knowledge in IR compare to other specialties as poor [16–18]. Furthermore, this gap between knowledge of IR and other specialties is widening over the years, indicating that students are making progress in their knowledge of other specialties but not in their knowledge of IR over their medical curriculum. Half of medical students considered that IR is surgery guided by imaging, which is accurate; indeed, a lot of minimally invasive surgeries and interventions are IR procedures, developed, refined, taught and safely performed by interventional radiologists whose clinical and anatomical expertise and specialty training in radioprotection and the utilization of complex imaging technologies enables them to deliver therapies for a wide variety of conditions throughout the body. Knowledge of the interventions performed by interventional radiologists is quite good, with at least two-thirds of the students being familiar with procedures performed in this specialty, whether vascular, oncologic or pain interventions. Overall, the reasons cited by students who indicated they do not have an interest in pursuing IR were dominated by three key points: concern with losing contact with patients (33%), lack of interest (~ 69.3%) and lack of knowledge (70.9%). We postulate that these three responses, and very probably the response to the previous question regarding interest in pursuing IR, might change substantially if IR were taught as an integral aspect of the medical curriculum across the entirety of medical studies.

We found it worth noting that the level of interest in IR by gender shifts based on the phase of medical study. During the preclinical phase, more women than men expressed interested in a career in IR; this tendency reverses in clinical phase, with more men than women interested in a career in IR. This reversal of interest deserves to be studied in more depth in order to understand why during their medical studies women lose interest in the specialty while men gain interest. Recent publications show that women could be discouraged from pursuing IR for various reasons (family charge, pregnancy, gender discrimination, sexual harassment) [19–21]. Moreover, despite the trend of increasing numbers of female medical students, women remain underrepresented in medical faculty and leadership positions [20]. This underrepresentation tends to reinforce itself: with a lack of female IR role models in academic appointments, women may well gravitate toward specialties better represented by female faculty, without real consideration for IR [22]. Hospitals, as long-standing institutions, have been slow to adapt to the shifts in social norms; institutions only change when the people who comprise them insist on change and implement it. Certainly, the barriers must be broken down. Academicians must be committed to encouraging female medical students to pursue specialties that best suit their talents and interests, regardless of current gender disparities in those specialties and advocate the development of working conditions in hospitals and other clinical environments that can support, and not restrict, the evolution of medicine into the future.

## Conclusion

New technologies and other advances, including artificial intelligence, are already changing, and will continue to change, the landscape of both diagnostic and interventional radiology. In this study, medical students expressed an interest in more information/lectures about IR during their medical curriculum, and nearly one quarter expressed interest in a career in IR. This is very promising for the future of the specialty. Further studies, involving broader groups across other geographies and investigating students’ end-point interests in diagnostic versus interventional practice would certainly help medical school faculty and operations to serve the demands of current students and plan for the future.

## Supplementary information

**Additional file 1**. Survey on radiology and interventional radiology.

## Data Availability

The datasets used and/or analyzed during the current study are available from the corresponding author on reasonable request.

## Abbreviations

AI: Artificial intelligence
CI: Confidence interval
IR: Interventional radiology
US: United States of America
