# Supplementary material for: Interventional radiology and artificial intelligence in radiology: Is it time to enhance the vision of our medical students?
Source: Insights Imaging. 2020 Nov 30;11:127. doi: 10.1186/s13244-020-00942-y (PMC7704872; doi:10.1186/s13244-020-00942-y)
Supplement: Supplementary file 1 — Additional file 1. Survey on radiology and interventional radiology. [file 13244_2020_942_MOESM1_ESM.docx]

**ELECTRONIC SUPPLEMENTARY MATERIAL**

**Survey on Radiology and Interventional Radiology**

This short survey is anonymous and seeks to determine the perception and knowledge of radiology and especially interventional radiology among medical students. You can answer in a few clicks on any support (mobile, tablet, computer) and click on "send" at the bottom of the page. Thank you very much for your participation and your valuable help. Diagnostic and Interventional Imaging Department

**Question 1. In which year of medical school are you?**

1^st^ year

2^nd^ year

3^rd^ year

4^th^ year

5^th^ year

6^th^ year

**Question 2. Are you?**

Female

Male

**Question 3. Do you think that Artificial Intelligence is a threat to Radiologists?**

Yes

No

**Question 4. Do you think Radiology has a future?**

Yes

No

**Question 5. Have you ever heard of the specialty of Interventional Radiology?**

Yes

No

**Question 6: If yes, where did you first hear of Interventional Radiology?**

From family, friends or patients

General reading

During a lecture

During a clinical attachment

**Question 7. Have you ever had a lecture in Interventional Radiology?**

Yes

No

**Question 8. Would you like more formal lectures and/or information on Interventional Radiology during your medical studies?**

Yes

No

**Question 9. How would you assess your knowledge of Interventional Radiology compared to other specialties?**

Excellent

Good

Adequate

Poor

No knowledge

**Question 10. Do you know how to become an Interventional Radiologist?**

Yes

No

**Question 11. Do you think Interventional Radiology is Surgery guided by imaging such as CT, MRI, ultrasound?**

Yes

No

Not sure

**Question 12. Among the following procedures, which are performed by an Interventional Radiologist:**

Pain treatment (musculoskeletal injections, tumor destruction, cementoplasty)

Tumor treatment (tumour destruction: bone, liver, kidney, lung)

Partial Nephrectomy

Thrombectomy (stroke)

Urgent and elective arterial embolization (road accident, post-partum bleeding, GI Bleeding, aneurysm...)

**Question 13: Would you be interested in a career in Interventional Radiology?**

Yes

No

**Question 14. If no, what are the reason(s)?**

Difficulty to obtain the specialty

Risk of radiation exposure

Lack of interest

Fear of losing contact with patients

Lack of knowledge
